# A large dataset covering the Chinese national sign language for dual-view isolated sign language recognition

**DOI:** 10.1038/s41597-025-04986-x

**Published:** 2025-04-19

**Authors:** Peng Jin, Hongkai Li, Jun Yang, Yazhou Ren, Yuhao Li, Lilan Zhou, Jin Liu, Mei Zhang, Xiaorong Pu, Siyuan Jing

**Affiliations:** 1 Sichuan Province Key Laboratory of Philosophy and Social Science for Language Intelligence in Special Education, Leshan Normal University, Leshan, 614000 China; 2 Key Laboratory of Internet Natural Language Intelligent Processing of Sichuan Provincial Education Department, Leshan Normal University, Leshan, 614000 China; 3 School of Computer Science and Engineering, University of Electronic Science and Technology of China, Chengdu, 611731 China

**Keywords:** Communication, Decision making

## Abstract

Isolated Sign Language Recognition (ISLR), which seeks to automatically align sign videos with corresponding glosses, has recently gained considerable attention from the artificial intelligence community. This technology has the potential to bridge the communication gap between hearing people and the deaf community. However, the development of ISLR is hindered by the scarcity of sign language datasets. Moreover, existing ISLR datasets are limited by their provision of a single perspective, which makes hand gesture occlusion difficult to handle. In addition, existing Chinese ISLR datasets, such as DEVISIGN and NMFs-CSL, fail to cover the entire vocabulary of Chinese National Sign Language (CNSL). This greatly obstructs the application of ISLR in the real world. To address these challenges, we introduce a novel word-level sign language dataset for ISLR that encompasses the entire CNSL vocabulary, comprising 6,707 unique signs. Moreover, it provides two perspectives of signers: the front side and the left side. There are ten signers involved in sign video recording, and the processes of sign video recording, annotation and quality assurance were rigorously controlled. To the best of our knowledge, this dataset is the first dual-view Chinese sign language dataset for ISLR that covers all the sign words in CNSL.

## Background & Summary

It has been reported that over 5 percent of the global population, approximately 430 million individuals, suffer from disabling hearing loss. In China, this number reaches approximately 30 million^1^. Sign language represents the primary mode of communication within the hard-of-hearing community. Many countries have developed their sign languages, such as Chinese Sign Language (CSL), American Sign Language (ASL), and British Sign Language (BSL). However, for hearing individuals, acquiring sign language is challenging due to its significant differences from spoken languages. Sign language is considered a visual language that employs multiple communicative channels, including manual and non-manual signals. Manual signals encompass hand shape, hand movement and pose, while non-manual signals involve facial expression, movement of the mouth, head, shoulders, and other gestures. Consequently, there is a critical need to develop effective tools to facilitate communication between the hard-of-hearing and hearing people, thereby bridging the communication gap.

Owing to advantages such as low cost and easy deployment, video-based sign language recognition (abbreviated as SLR) has attracted considerable attention from the artificial intelligence community. SLR can be categorized into two groups, i.e., isolated sign language recognition (ISLR) and continuous sign language recognition (CSLR), which aim to translate a sign language word or sentence into a gloss or a gloss sequence, respectively. Sign language translation is a task similar to CSLR, and it continues to adjust the order of gloss sequences to make the results easier for hearing people to understand. Owing to the rapid development of deep learning techniques, the recognition accuracy of SLR has improved significantly. Camgöz *et al*.^2^ proposed an end-to-end SLR model, which, to our knowledge, is the first work that introduces a deep neural network (DNN) into this field. Researchers subsequently proposed different DNN models for improving the performance of SLR, such as hybrid DNN models^3–7^, multicue DNN models^8^, and transformer-based models^9,10^. In 2021, the CVPR conference, which is the most famous conference in the field of computer vision, held a competition for ISLR worldwide^11^. The winning team^12^ proposed a multimodal model for ISLR that achieved a score of 0.984 for the top 1 accuracy on the AUTSL dataset (https://cvml.ankara.edu.tr/datasets)^13^. In addition, Coster *et al*.^14^ proposed another method that utilizes the pose flow of the signer as well as a self-attention mechanism. Owing to the lack of large-scale datasets for SLR, researchers have explored several advanced DNN techniques to improve performance, such as pretrained models^15–17^, auto- and non-auto- regressive decoders^18,19^, reinforcement learning^20^, contrast learning^21^, and other models^22,23^.

However, the development of SLR is also hindered by the scarcity of large-scale datasets. Although some works have introduced effective techniques such as transfer learning^24^ and data augmentation^16^ to mitigate this problem, the training of effective deep neural network (DNN) models for SLR necessitates substantial datasets. Despite sign language being a low-resource language, which complicates the collection of adequate data for training DNN models, the academic community has contributed several significant datasets. For example, there are a number of popular datasets for ISLR, such as WLASL (https://dxli94.github.io/WLASL/)^25^, MSASL (https://www.microsoft.com/en-us/download/details.aspx?id=100121)^26^, DEVISIGN (http://vipl.ict.ac.cn/homepage/ksl/data.html)^27^, NMFs-CSL (https://ustc-slr.github.io/datasets/2020_nmfs_csl/)^28^, and AUTSL^13^. However, these datasets present two deficiencies that impede the application of ISLR techniques in real-world settings. First, the existing datasets for ISLR typically offer only a single perspective, which does not adequately address the issue of hand gesture occlusion. This limitation significantly affects the accuracy of sign language recognition. Second, for Chinese ISLR, the available datasets, such as DEVISIGN and NMFs-CSL, do not encompass the whole vocabulary of CNSL^29^, limiting their utility for comprehensive language recognition applications. These gaps underscore the need for enhanced dataset development that not only broadens the size of sign language vocabularies but also incorporates multiple perspectives to effectively capture the full range of sign language gestures.

To address the challenges outlined previously, this paper introduces a new dataset for the ISLR task, which is named the NationalCSL-DP (National Chinese Sign Language dataset with Dual Perspectives). The dataset is distinguished by several notable features:

- Extensive Vocabulary: The dataset consists of 6,707 distinct sign motions as well as 134,140 sign videos, surpassing the vocabulary size of existing datasets. For example, WLASL, which is a large-scale dataset for ISLR, contains only 2,000 different sign motions. This extensive collection enables more comprehensive training and testing of ISLR models.
- Dual Perspectives: The dataset provides two perspectives for each sign motion: the front side and the left side. This dual-perspective approach aims to mitigate the issues related to hand gesture occlusion, thus improving the accuracy and robustness of ISLR models. To our knowledge, How2Sign^30^ is a unique dual-perspective sign language dataset for CSLR. However, there is no dual-perspective sign language dataset for ISLR.
- Rigorous Validation: We performed meticulous manual checking to ensure the high quality of the dataset. Moreover, a technical validation was also carried out to evaluate its quality. This rigorous validation process guarantees the dataset’s reliability for training effective ISLR models.

By leveraging the dataset, we aim to significantly advance the development of ISLR techniques, thereby facilitating improved communication between the hard-of-hearing community and hearing people. This dataset is expected to be a valuable resource for researchers and practitioners working to bridge communication gaps through enhanced sign language recognition capabilities.

## Methods

The participants were recruited from the undergraduate students of Leshan Normal University (LSNU). All participants have provided informed consent forms for the sharing of their identity information and signed agreements to consent to participate in the construction of the dataset NationalCSL-DP, as well as to allow the dataset to be published, including but not limited to academic journals and online databases. The Ethical Review Board (ERB) of LSNU reviewed our ethical review application, as well as the informed consent forms and agreements of all participants regarding the sharing of identity information as well as the dataset publication, etc. Finally, permission was granted by the ERB of LSNU for the open publication of the dataset, including the manuscript submission and the dataset release (Ethical Review Number: LSNU-KYLL2025-02-15).

### Participants

Ten participants (2 males and 8 females, mean age 19.82 ± 0.28SD years) were recruited for dataset development. These participants, consisting of 8 deaf students and 2 hearing students, are all proficient in CNSL. They were in charge of three tasks. First, they excluded some sign words with the same sign motion from the CNLS (e.g., “战争”(war), “战斗”(combat), “战役”(campaign)) to ensure that the motion of the sign words in the dataset was distinct. Second, they recorded videos of all the sign words in the vocabulary list. In this step, at least one sign language expert was on site to supervise the video recording. After that, all the participants annotated the sign video; i.e., they assigned a gloss to a sign video. Because this annotation process is error prone (which will be explained later), they performed cross-verification to ensure the correctness and quality of the dataset. In addition to these steps, we also performed technical validation to ensure the accuracy and comprehensiveness of the dataset. The collection and public sharing of data in this study have obtained the consent of all participants, and all of them have signed the authorization agreements.

### Description of CNSL

**Fig. 1:**
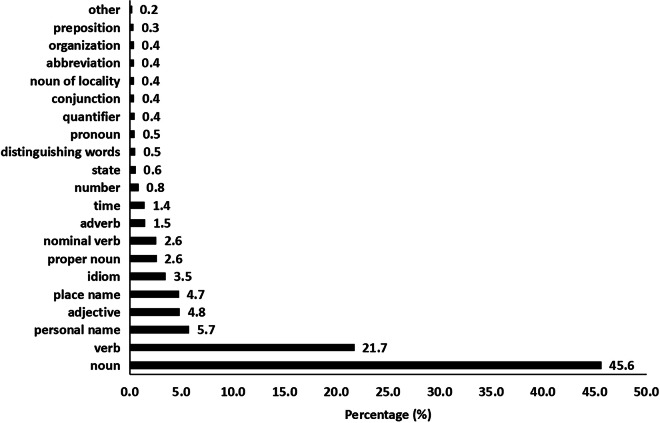
A statistic of the clipped vocabulary list from CNSL.

The latest CNSL was officially released in 2018 and contains 8214 sign words^29^. There are three types of sign words that should be considered in CNSL. The first type is the sign words that are different but have the same hand motion, e.g., “保护” (protection) and “维护” (maintenance). For these words, we excluded them and kept the first one according to their order in the CNSL vocabulary. Finally, there are 6707 sign words in the clipped vocabulary list. The statistics of the vocabulary list are shown in Fig. 1. These statistics were calculated based on the jieba toolbox, in which the words are divided into 35 categories, such as nouns, verbs, and adjectives. The figure shows only the categories in which the word frequency is greater than 30. The second type is the sign words that have the same gloss but different meanings and hand motions, e.g., “安全带” (safety belt) (as shown in the figure below). The figure on the left represents the safety belt in an aircraft, and the figure on the right represents the safety belt in a car. To distinguish them, we added a suffix “1-X” after the original gloss label, such as"安全带1-1” and “安全带1-2”. The third type is the sign words that have the same meaning and gloss but with different hand motions, e.g., “政府” (government). We add the suffix “2-X” after the gloss label to distinguish this type of word. For example, “政府” was annotated to “政府2-1” and “政府2-2”, which correspond to the sign language “government” in northern China and southern China, respectively.

Both manual and nonmanual features are important for sign language recognition and sign language machine translation. The former includes hand gestures, hand movements, and hand orientations. The latter includes body posture, facial expression, and other postures. Some studies believe that facial expressions are helpful only for CSLR and sign language machine translation (SLMT) but are trivial for word-level sign language recognition, i.e., ISLR. In fact, some sign words in the CNSL are asked for facial expressions, such as “是……还是……” (the word “or” in English), which emphasizes the transposition of the head. In addition, most of the sign words indicating mental action and the state of things depend on the facial expression of the signers. Therefore, focusing only on manual features and ignoring nonmanual features leads to a reduction in recognition accuracy.

### Experimental procedures

In this section, we explain the procedure of the development of the sign language dataset. Because facial expression is an important nonmanual feature in sign language, we signed a copyright with every signer to determine the ownership of the portraiture right of the sign videos in the dataset. In other words, the dataset released in this paper is released under the conditions of noninfringement.

**Fig. 2:**
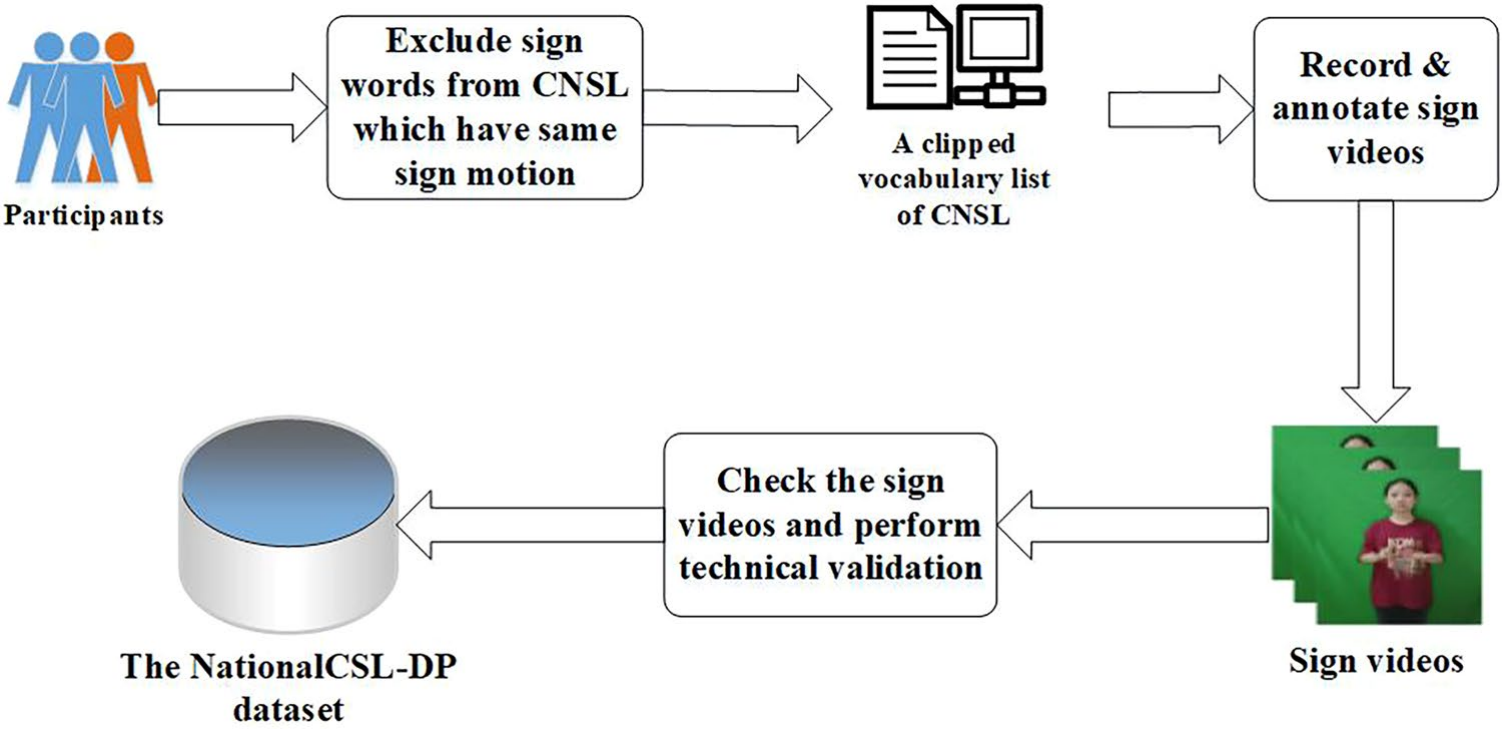
The process of dataset development (the participant consented to the open publication of the image).

A flowchart for describing the development of the sign language dataset is shown in Fig. 2. Generally, the process of dataset development consists of three stages. First, we excluded sign words with the same sign motion and outputted a clipped vocabulary list. The vocabulary contains the sign words that are recorded in the next step. Next, all the participants signed these words in a standard environment. The sign videos were recorded and annotated by the signers themselves. After that, a rigorous cross-check was conducted to ensure the quality of the dataset.

**Fig. 3:**
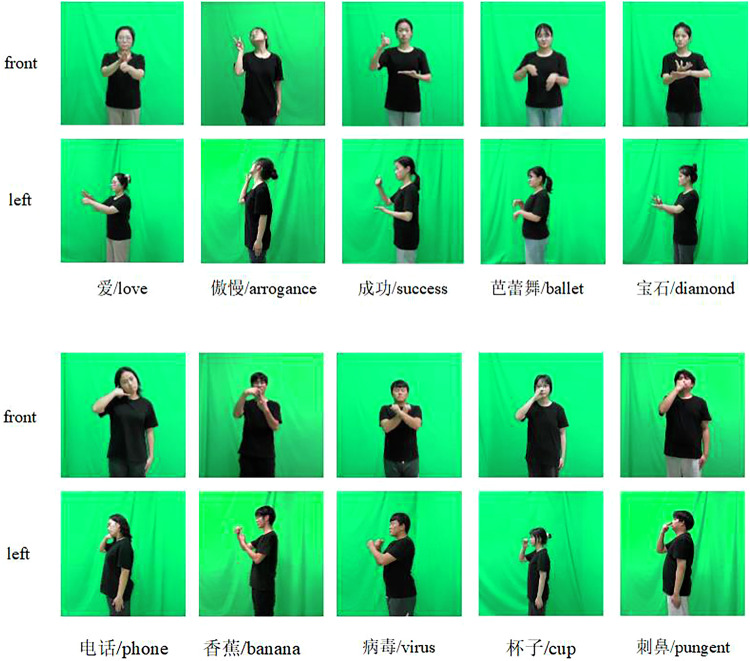
Examples of sign videos (the participants consented to the open publication of the image).

Video recordings were performed in a supervised setting at two green screen studios. Each green screen studio was equipped with two high-definition cameras, which were placed on both the frontal side and the left side of the signer. Specifically, when a signer is signing, the two cameras record synchronously. All the cameras recorded videos at 1920 × 1080 resolution at 50 fps. Samples of the data recorded in this study are shown in Fig. 3. Each sign word was performed by ten signers. During video recording, there were two assistants, a signer and an expert in sign language. The assistants were in charge of camera operation, and the expert supervised the motion of the signer to ensure the quality of the videos. All the signers were asked to wear black T-shirts. They were instructed to put their arms naturally on their sides at the beginning and end of the video. Moreover, the body above the knee, especially the arms, of the signers needed to remain within the video range at all times. To improve recording efficiency, each signer recorded 100 sign words at a time. After that, each video was cut into 100 clips, each of which matched a sign word. However, video editing and annotation are tedious tasks; therefore, many errors can occur at this stage. More details will be introduced in the next section. Because all the signers were students in school, i.e., they could record sign language videos only in their spare time; the sign video recording process took approximately 5 months.

### Annotations

The task of annotation is to assign a gloss label to each sign video clip, which corresponds to a sign word in CNSL. We have explained that the number of sign language words is 6707 and that each sign word was performed by ten signers with two perspectives, i.e., the front side and the left side. Therefore, 134140 sign videos were ultimately obtained. The total duration of the sign videos is over 88.32 hours. Editing and annotating sign videos is time-consuming, and the process is prone to errors. To ensure the quality of the dataset, a cross-checking step was carried out by all the participants. They checked the sign videos carefully and identified seven kinds of problems. This checking process took approximately 45 days, and 3068 problems were identified. The details of the problems are explained below.

- Annotation error. There were two different causes for errors. The first cause was typos. For example,“泄漏”(leak) was incorrectly labeled as “泄露”, “津巴布韦”(Zimbabwe) was incorrectly labeled as “津巴韦布”. The second cause of annotation errors was that the glosses of several adjacent videos were shifted; i.e., each video was incorrectly annotated to the next gloss in the vocabulary list.
- Motion error. Although we invited sign language experts to supervise the video recording, there were still some motion errors in which the signers incorrectly performed the sign word.
- Editing error. Some videos were carelessly edited; thus, they contained inappropriate content, such as stretch, drink water, chat, or other types of unnecessary information.
- Poor quality video. Issues included video stabilization, black screens, and corrupt video files.
- Background problem. Some videos were invaded by unnecessary objects, such as a basketball and LEDs.
- Inappropriate recording. In most cases, this occurred because the HD camera was too close to the signer; thus, the hand or arm of the signer exceeded the range of the video.
- Missing. Every signer was asked to complete 6707 front-side sign videos and 6707 left-side sign videos. However, some videos were repetitive. This may have been caused by incorrect editing or copying.

## Data records

**Fig. 4:**
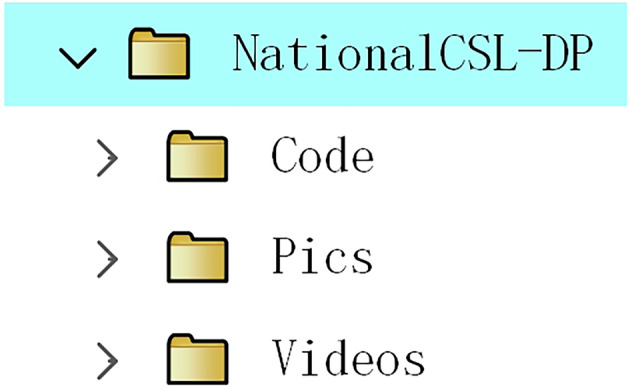
The structure of the dataset folder.

The data collected are available at Figshare^31^https://figshare.com/articles/media/NationalCSL-DP/27261843. As shown in Fig. 4, there are three folders in our dataset: “Videos”, “Pics”, and “Code”. The “Videos” folder contains the raw sign videos recorded by ten signers with two perspectives, i.e., the front side and the left side. The resolution of the raw sign video is 1920 × 1280. Since the size of the raw sign videos exceeds 1.8 TB, due to space limitations, only 1 percent of the raw sign videos were uploaded. The raw data is available at http://lise.lsnu.edu.cn/kxyj/dbxcg2.htm. The “Pics” folder provides the image frames extracted from the raw sign videos, which were scaled to a resolution of 256 × 256. The image frames of all the sign videos were uploaded to this folder. Finally, the code used to generate these images can be found in the last folder. More details about these folders are explained below.

### "Videos” folder

**Fig. 5:**
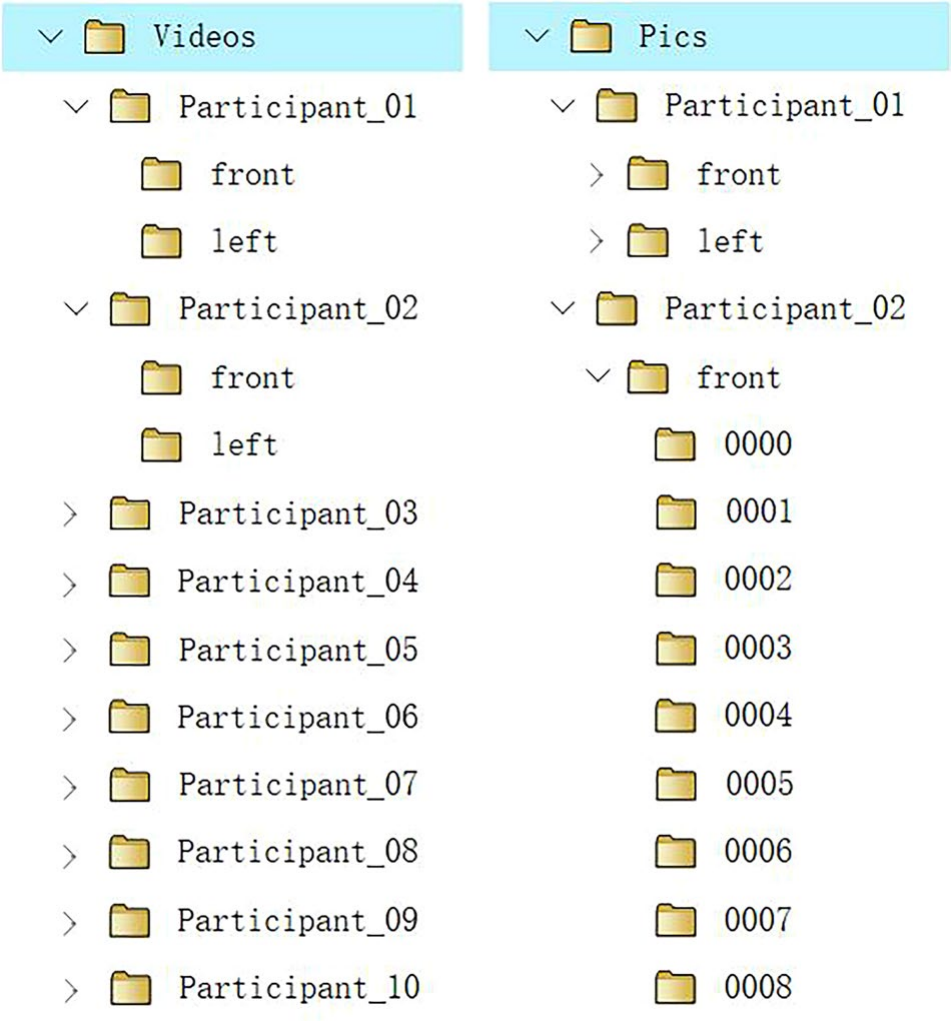
The structure of the “Videos” and “Pics” folder.

The “Videos” folder contains 10 subfolders. Each subfolder corresponds to a unique signer and provides approximately ten percent of the raw sign videos recorded by the signer. The name of each subfolder is Participant_01, Participant_02, etc., as shown in Fig. 5 (left). Every sign word in the vocabulary list was performed by ten participants, and we recorded the sign videos from two perspectives, i.e., the front side and the left side. Therefore, in each subfolder, there are two additional folders named “front” and “lateral”, which store the videos from the frontal and left perspectives, respectively. Each raw video corresponds to a word in the vocabulary list, and each video is assigned a unique number which corresponds to its index in CNSL. Moreover, the corresponding gloss (both in Chinese and English) can be found in a meta file named “gloss.csv”. Notably, owing to the use of different recording devices, there are two different file suffixes for the raw sign videos, which are “MTS” and “MP4”. The resolution of all sign videos is 1920 × 1680.

### "Pics” folder

**Fig. 6:**
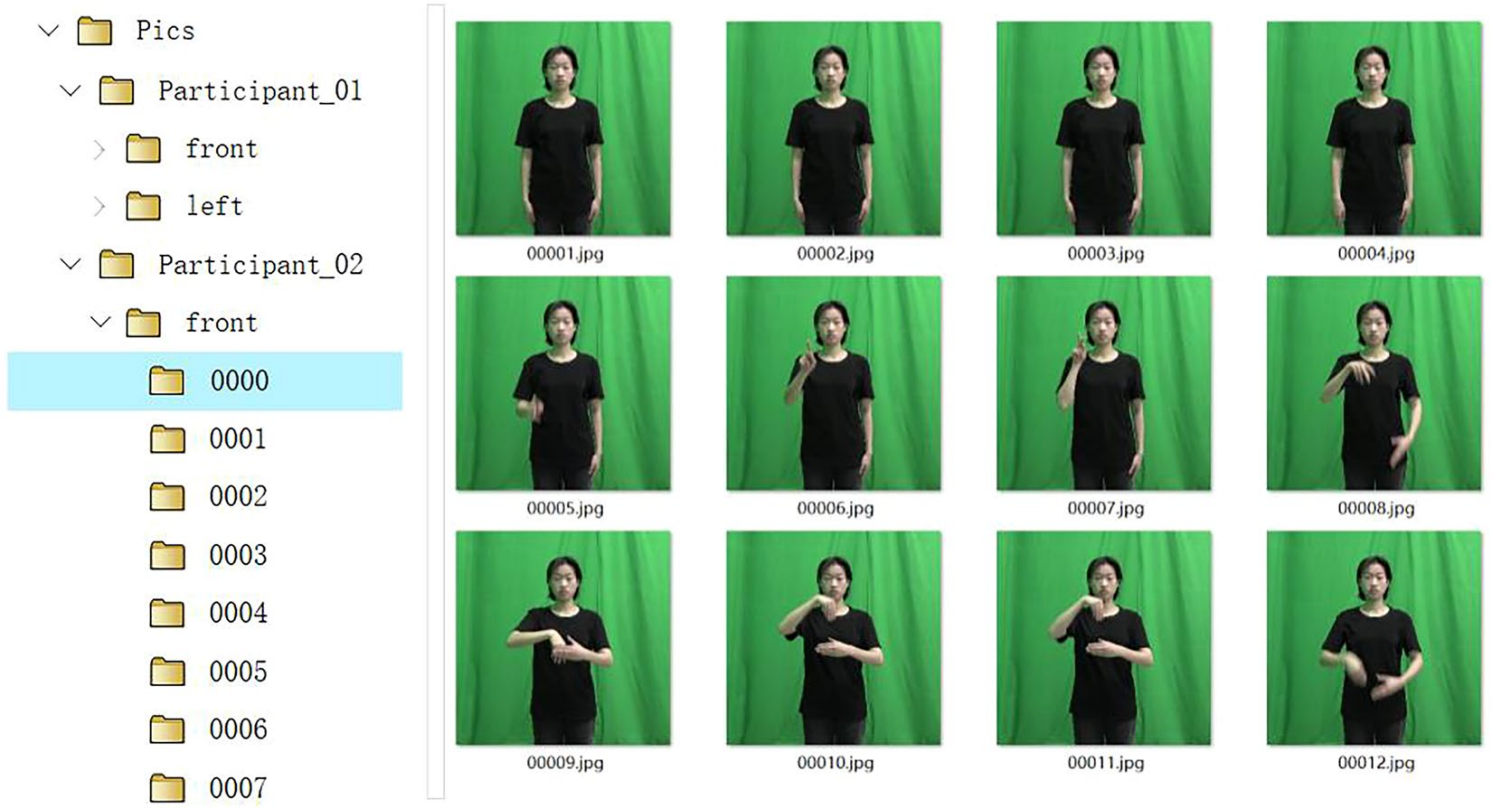
Image files in the “Pics” folder (the participant consented to the open publication of the image).

The “Pics” folder stores image frames extracted from the raw sign videos. Like the structure of the “Videos” folder, it also contains 10 subfolders, each of which corresponds to a participant, as illustrated in Fig. 5 (right). Like the “Videos” folder, each subfolder contains two additional folders that provide the image frames from the front side and the left side. The difference is that, in the “Pics” folder, each sign word has several image files that are sampled at a fixed rate from the raw sign videos of both perspectives. These image files were named according to their order of appearance, such as 00001.jpeg, 00002.jpeg, and 00003.jpeg (as shown in Fig. 6). In addition, we performed central cropping on the images to maintain equal pixel dimensions in both the horizontal and vertical directions. The resolutions of all the image files were scaled to 256 × 256. More explanations can be found in Table 1.

**Table 1:** Explanation of the folder structure of “Pics”.

Folder structure	Example	Explanation
1st level	CSY, CXY, ……	Abbreviation of signer’s name
2nd level	front, left	Different views of the signer
3rd level	扁担,饼, ……	The gloss of the sign words. 扁担 = carrying pole 饼 = flatbread
4th level	00001, 00002, ……	Frames extracted from the video. The numbers represent the sequence of frames.

### "Code” folder

**Fig. 7:**
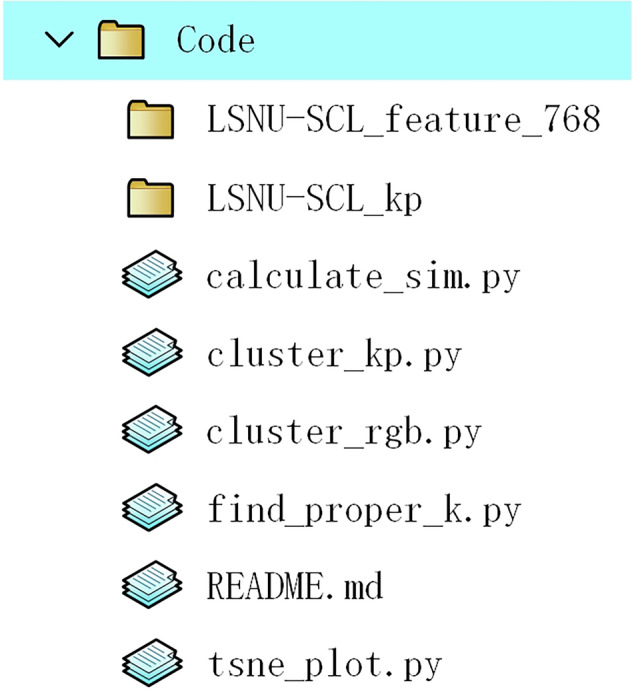
The structure of the “Code” folder.

The “code” folder consists of two parts. The first is the code for converting the raw sign video into frame images, which is implemented in Java. The JavaCV https://github.com/bytedeco/javacv library is necessary to run the code correctly. Moreover, it employs the OpenCV library for video processing, e.g., for reading frames from the sign video and saving a frame as a JPEG file. The other is for technical validation that provides RGB features and Skeleton features extracted from the dataset, five Python scripts for cluster analysis, and a README guide (see Fig. 7). For detailed introductions to the code and usage instructions, please refer to the README. For feature extraction, we used the Swin Transformer^32^ and SL-GCN^12^ as our backbone models, and we also utilized the open-source toolkit scikit-learn^33^. Next, we will explain the technical validation in detail.

## Technical Validation

To verify the quality of the sign language dataset, we conducted cluster analysis on the dataset and calculated the Intraclass Pose Similarity (IPS) and Intraclass Video Similarity (IVS) to assess the effectiveness of the clustering. Through cluster analysis, we identified the samples with the lowest similarity with the same gloss label; these samples tended to be assigned to different clusters during clustering. We also discovered samples with different gloss labels but the highest similarity, which are often grouped into the same cluster during the clustering process. Subsequently, we randomly selected 5% of these samples for human verification and analysis to ensure that the clustering results aligned with human judgment in terms of accuracy and consistency.

### Cluster analysis

**Fig. 8:**
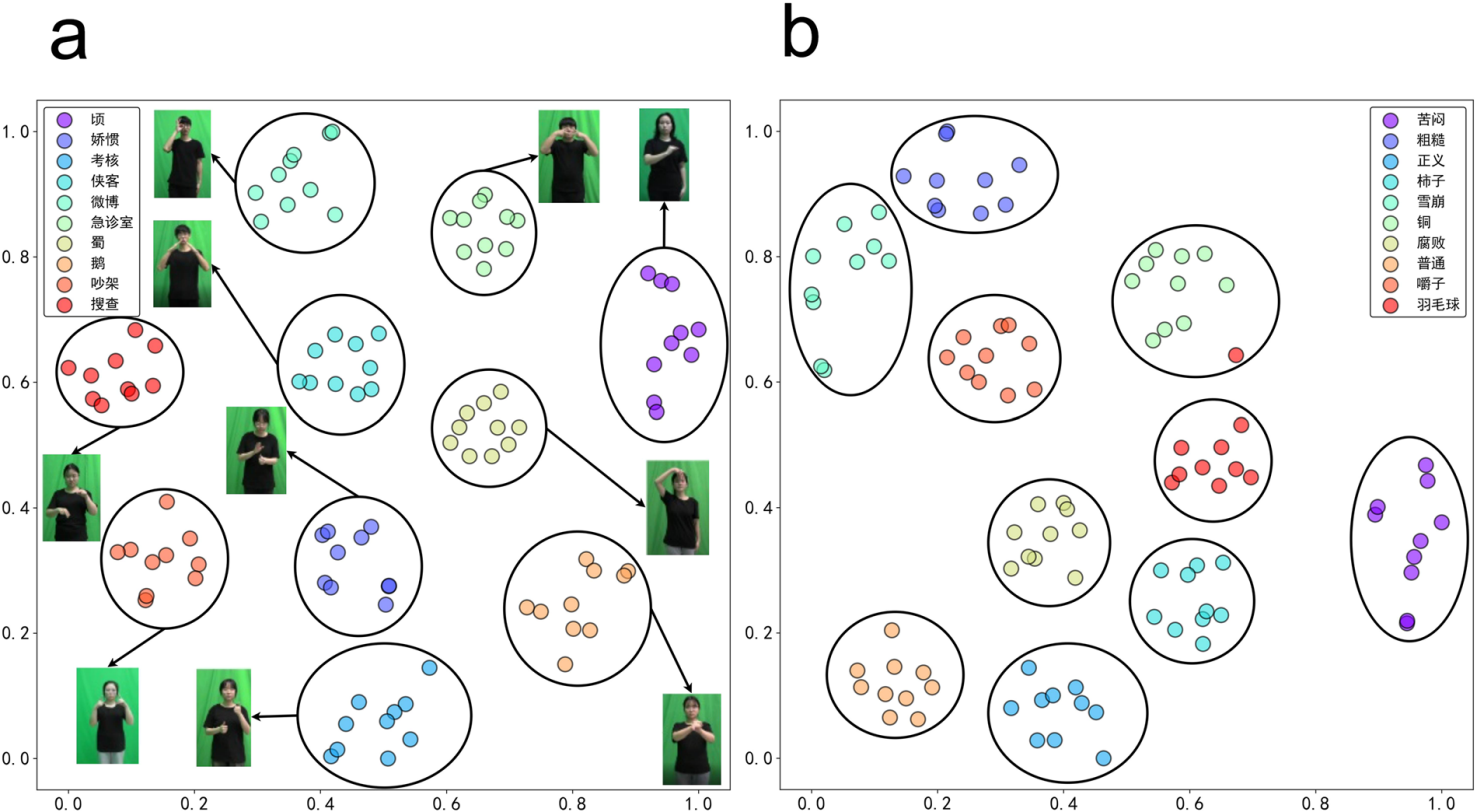
The t-SNE visualization of the extracted feature maps for the RGB and joint modalities. (**a**) RGB feature map. (**b**) Joint feature map. (the participants consented to the open publication of the image).

Before conducting cluster analysis, we trained a video transformer encoder on the proposed sign language dataset. Then, we used the trained encoder to encode sign language videos, converting them into 768-dimensional feature vectors (encoding skeleton data into 256-dimensional feature vectors). We subsequently utilized t-Distributed Stochastic Neighbor Embedding (t-SNE)^34^ for dimensionality reduction and visualization of the feature maps of the sign language videos. As shown in Fig. 8a, in low-dimensional spaces, these feature maps maintain good discriminability, which validates the feasibility of clustering sign language video data and provides us with an effective approach to explore and understand the intrinsic structure of sign language data.

Finally, we performed *K*-means clustering on this reduced dataset. As shown in Table 2, we employed five common clustering evaluation metrics, including accuracy (ACC), Adjusted Rand Index (ARI), Normalized Mutual Information (NMI), F1 score, and V-Measure. A higher NMI value indicates that the clustering result is more similar to the true partition. The values of NMI and V-Measure are relatively high, indicating that the clustering results perform well in terms of information sharing and consistency. This suggests that the samples within the same cluster exhibit high similarity, and there is good differentiation between samples from different clusters. However, the relatively low values of the ARI and ACC indicate that the clustering results do not align well with the true labels, suggesting that some samples may have been misclassified.

**Table 2:** The clustering results of RGB data and skeleton data.

modality	*K* = 6707				
	ACC(%)	ARI(%)	NMI(%)	F1(%)	V-Measure(%)
RGB	41.40	23.43	83.31	40.73	83.31
Skeleton	41.08	23.07	83.06	39.85	83.12

### IPS and IVS

By examining the average similarity within clusters, we can gain insights into the consistency within clusters and identify reasons for relatively low ACC and ARI scores. High similarity scores typically indicate that samples within a cluster are very similar to each other, suggesting strong consistency within the group. Conversely, low similarity scores may reveal diversity or heterogeneity within the cluster, indicating greater variability among samples.

As shown in Fig. 8b, a sample in “羽毛球” (badminton) category has greater similarity to samples from different categories. Additionally, we found that the sizes of the clusters in the clustering results are not balanced, with some samples being assigned to other categories of gloss. As a result, the clusters in the clustering results do not correspond to the true gloss labels, resulting in relatively low values for ACC and ARI. Ultimately, we identified 243 outlier samples by analyzing intragroup similarity.

**Fig. 9:**
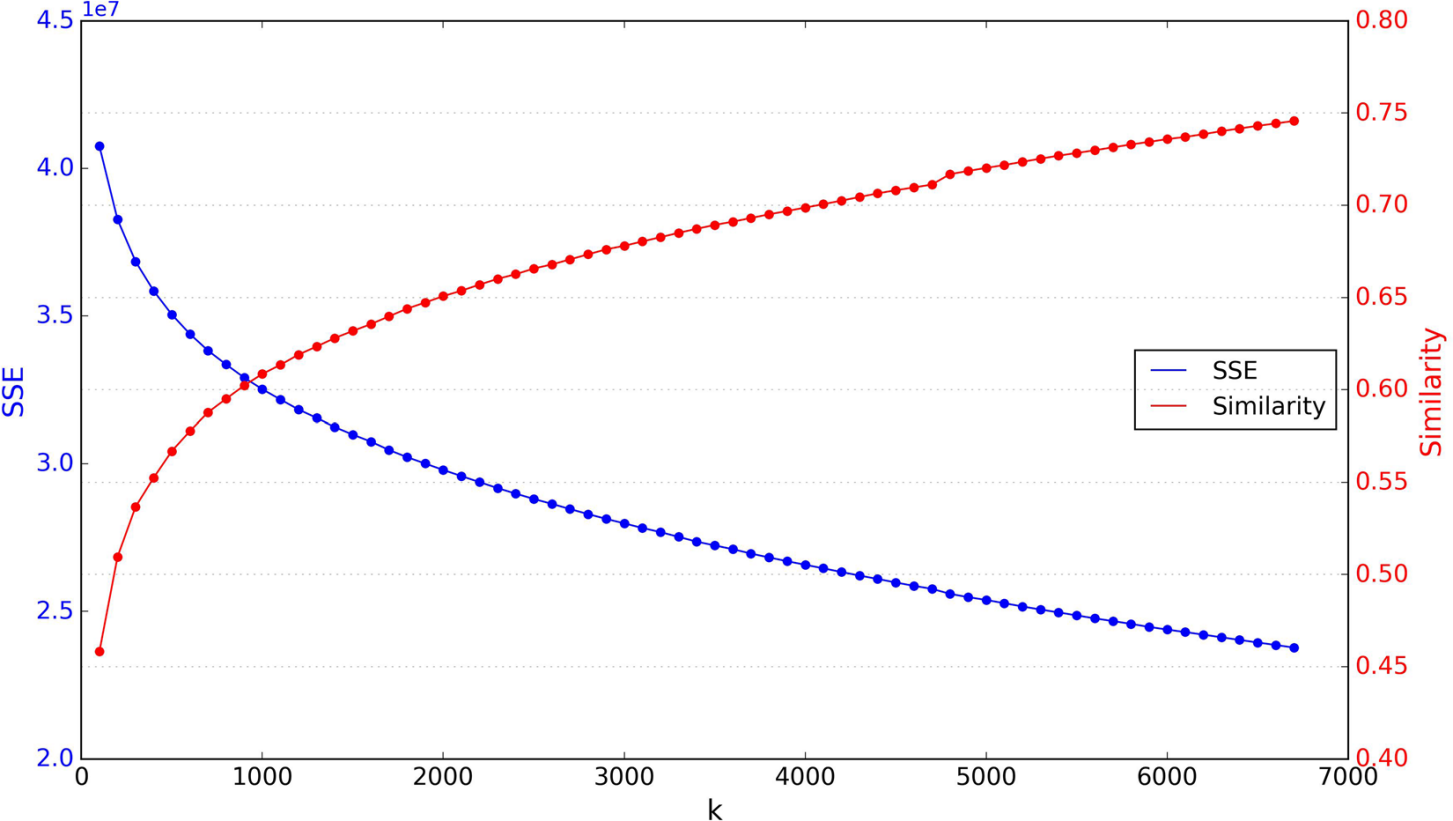
The impact of the number of *K* clusters on intraclass similarity and SSE.

As shown in Fig. 9, we calculated the impact of different numbers of *K* clusters on the intraclass similarity and the sum of squared errors (SSE). To achieve the smallest SSE and the highest intraclass similarity as much as possible, we should increase the number of *K* clusters. The number of categories in our dataset, 6707, is a relatively good number of *K* clusters, which also indirectly confirms the strong separability of our dataset.

### Human check

After conducting cluster analysis, we performed a manual check to verify the clustering results. We randomly selected 5% of the outlier samples for manual review, specifically those that belonged to the same category but had very low similarity and those that belonged to different categories but had very high similarity. During the manual check process, videos were assessed based on the clarity of gestures, the accuracy of gesture expression, and consistency with the sign language dictionary. We found that intraclass similarity from the cluster analysis could identify samples with naming errors and those with video image is sues. Among the 243 detected outlier samples, a total of 182 samples were found to be anomalous, with 153 sample naming errors and 29 video image issues. The recall rate reached 94.14%, and the accuracy rate also reached 74.89% when intraclass similarity was used to detect issues in the dataset, which significantly reduced the workload of manual inspection. In other words, the introduced technical validation method can not only prove the effectiveness and quality of the dataset but also provide a tool for detecting errors in the dataset, which can significantly alleviate manual error checking.

## Data Availability

The codes for data preprocessing and technical validation are available in the subfolder named “Code” under the root at^31^https://figshare.com/articles/media/NationalCSL-DP/27261843.
